# Editorial: Nutrition and Management of Animals We Keep as Companions

**DOI:** 10.3389/fvets.2021.748776

**Published:** 2021-09-28

**Authors:** Anna K. Shoveller, Guido Bosch, Luciano Trevizan, Joseph J. Wakshlag, Daniel A. Columbus

**Affiliations:** ^1^Department of Animal Biosciences, University of Guelph, Guelph, ON, Canada; ^2^Department of Animal Sciences, Wageningen University and Research, Wageningen, Netherlands; ^3^Department of Animal Science, Universidade Federal do Rio Grande do Sul, Porto Alegre, Brazil; ^4^Department of Clinical Sciences, Cornell University College of Veterinary Medicine, Ithaca, NY, United States; ^5^Prairie Swine Centre, Inc., Saskatoon, SK, Canada

**Keywords:** pet food, nutrition, canine, feline, nutrition, digestion, metabolism, health

## Summary and Commentary

Pet ownership has recently grown at an unprecedented rate, with The Association of Pet Products Manufacturing Association (APPMA) (1) reporting that pet ownership has increased 3% in the last year, with an estimated 70% of U.S. households owning at least one pet. The APPA also reports that pet owners also increased spending and, largely due to the COVID pandemic, shifted where they buy food with direct-to-consumer purchasing on the rise, with 86% of pet owners purchasing pet food online in 2020, up from 72% in the previous year. Most recently, the millennial generation has now taken over as the largest pet-owning generation, followed by boomers, then GenX. While the growth of the pet food industry is partly driven by ownership, there is also growth stimulated by the growing appreciation of the value of pets to human health and well-being. Indeed, growing evidence suggests physical and mental benefits resulting in positive health outcomes for pet-owning humans. As our bond with companion animals grows, we need to support their role in our lives by better understanding the effects of nutrition, and other parts of the environment that we share with them, on the metabolism, health, and well-being of our companion animals. This special issue adds to the primary literature concerning canine and feline metabolism, nutrition, and behavior.

With a growing global human population and increased demand for food, there is a need to identify novel or alternative food ingredients and processes for pet foods that increase nutrient availability. These ingredients may replace ingredients that can be used in the human food sector, reducing the competition for resources across the food chain. Physical, chemical, or thermal processes that improve digestion and nutrient availability can improve digestion and nutrient delivery to the organism resulting in a reduced need for overall food intake. The food format most commonly used for companion animals is extrusion and as such, cereal grains are widely used in pet food to supply carbohydrates, but also to support optimum physical kibble characteristics. However, dietary carbohydrates are associated with a post-prandial blood glucose response, and ingredients that elicit a large glycemic response have been implicated in weight gain and obesity. Teixeira et al. found the partial replacement of corn with sorghum and with and without hydrolysable tannin extract did not elicit differences in glycemic response, total tract apparent digestibility of dry matter, organic matter, and macronutrients among treatments. These data help to support the inclusion of sorghum instead of corn. Similarly, von Shaumburg et al. examined the inclusion of white and red sorghum grains on the gastrointestinal health of cats and found that both sorghum ingredients were well-tolerated by cats and, for the most part, did not differ from the control that included corn. These data further support the inclusion of these ingredients. Reilley et al. added to our available ingredients with the investigation of legumes (garbonzo beans, green lentils, peanut flour) and yeast, and all in contrast to poultry by-product meal on palatability and digestibility in adult dogs. The inclusion of plant-based proteins did not affect apparent total tract digestibility of macronutrients, yeast increased fecal branched-chain fatty acid, fecal butyrate concentrations, and total fecal short-chain fatty acid concentrations. Yeast fed dogs also had greater β-diversity. As such, the researchers concluded that all ingredients are potential ingredients in canine diets and may support beneficial shifts in the microbiome. Similarly, but for the purpose of providing dietary fiber, Finet et al. examined the novel fiber source miscanthus grass, comparing it to beet pulp + tomato pomace and cellulose. While cats fed beet pulp had the greatest concentration of short-chain fatty acids, with the exception of butyrate, which was similar, the cats fed miscanthus grass has greater α-diversity than cats fed beet pulp. Overall, these data suggest that miscanthus grass fiber and fiber blends are good alternatives to the commonly used dietary fiber sources such as beet pulp and cellulose. The research into alternative ingredients and how to appropriately allocate these across the food chain will be integral to the future sustainability of the pet food industry and impact our ability to produce high nutritional quality foods, and we will continue to see investment in this area of research.

The option of diet formats is also increasing in propensity and indeed, raw meat-based diets have been touted to improve metabolic health, but little data exists to support these assertions. Moore et al. compared serum metabolites in client-owned Staffordshire bull terriers with canine atopic dermatitis and fed either a raw meat-based diet (RMBD) or a kibble. Dogs fed kibble had greater concentrations of methionine than dogs fed RMBD, but lower concentrations of carnitine and creatine. The dogs with atopic dermatitis experienced a larger metabolic shift in response to diet, but diet did not change the severity of canine atopic dermatitis. Building on those outcomes, Antuaniemi et al. sought to understand how the skin transcriptome is affected by atopic dermatitis and the feeding of raw diets in contrast to kibble. At the end of feeding a median of 137 days of one of these dietary treatments, 149 genes were found to be differentially expressed in transcripts between atopic and healthy dogs, and all genes were upregulated in the raw fed dogs in contrast to the kibble dogs. Interestingly, atopic dermatitis was largely associated with alterations in lipid and keratinocyte metabolism and angiogenesis. Further, the transcriptome in raw-fed dogs suggests enhanced innate immunity and lower oxidative stress and provides further support for this format of feeding. However, a key obstacle to feeding raw diets is understanding the nutrient content of those ingredients to enable precise food formulation based on nutrient targets of the final product. One feeding format that is being increasingly used to feed domestic cats is whole-prey diets, a version of raw meat-based diets. Owens et al. measured the crude protein, moisture content, and amino acid concentrations in fresh and frozen ground rabbits with or without gastrointestinal tracts (GIT). Focusing on the indispensable amino acids, fresh rabbits with GIT had greater concentrations of methionine, lysine, histidine, and arginine, and freezing resulted in lower methionine in the ground rabbit without the GIT. Most importantly, all indispensable amino acids exceeded recommendations of regulatory agencies except for taurine, which was below recommendations and suggests that taurine may need to be provided to cats if consuming a whole prey diet based on rabbit. Taken together, raw meat-based diets have a place in our feeding management of companion animals, but we need more data on not only the effects of feeding these diets, but the characterization of raw ingredients to support product development.

A key concern for diet management is the effect of early nutrition on the onset of disease later in life and diet format results in a different dietary chemical profile that may be a significant determinant. Hemida et al. investigated the effect of early life dietary, environmental, demographic, and genetic variables and whether these factors are associated with inflammatory bowel disease in dogs. Interestingly, and in opposition to current recommendations for pets, this cross-sectional questionnaire-based study suggested that feeding a high fat, low carbohydrate, non-processed meat-based diet resulted in significantly less inflammatory bowel disease later in life than when an ultra-processed, carbohydrate-based diet was fed. Not surprisingly, a normal body condition early in life supported a lower incidence of IBD, while greater body condition scores were related with a greater prevalence of IBD in dogs. Along the same vein, dietary transition is commonly reported to cause gastrointestinal distress and numerous dietary solutions are being investigated, of which yeast products could hold promising bioactivity. Lin et al. found that supplementing a yeast cell wall fraction supplement tended to result in greater *C. perfringens* and a rapid diet transition to a canned diet or a high fiber diet did not alter fecal pH, dry matter, calprotectin, and *E. coli*, but the inclusion of yeast during this dietary transition tended to result in greater fecal IgA. While a subtle change, the greater IgA suggests enhanced intestinal immune function, and as such, yeast products may help to support gastrointestinal health after dietary transition. Diet change is not the only thing that may disrupt the gastrointestinal environment, high-intensity exercise may also challenge this environment and dietary fiber optimization was studied in a study by Thornton et al. Sled dogs were fed diets containing kibble with a 4:1 or 3:1 insoluble:soluble fiber ratio and also had increasing amounts of exercise over a 9-week period. When sled dogs received the 3:1 diet they experienced greater fecal short-chain fatty acids and a lower internal body temperature than dogs who received the 4:1 but experienced no differences in body composition or respiratory rate. Separately, after the period of step-wise increases in exercise, dogs had an average 7% increase in lean and a 3.5% decrease in fat mass. With sporting dogs, an improvement in the soluble fiber delivery may improve gut health and will not impact performance and indeed, exercise, albeit higher intensity than most pet dogs would receive, does produce favorable shifts in body composition and we need to consider appropriate exercise for our dogs and cats to support optimum metabolism.

There has been recent interest in how diet affects cardiac health in dogs and a suggestion that the inclusion of legumes or elimination of grains may underpin the increased reporting of dilated cardiomyopathy in particular. Reis et al. examined the inclusion of fava beans in a moderate protein diet and compared that to two commercial diets, one with normal protein (25% crude protein and containing grains) and high protein (41% crude protein containing no grains) and largely using fava beans as a dietary protein ingredient. Similar to Teixeira et al. high-tannin and fermentation were also included in the design. There are a myriad of differences between the treatments of high protein and low protein and high tannin and low tannin, but interestingly, dogs fed normal protein for 7 days experienced greater left ventricular end-systolic volume and cardiac output than dogs fed high protein, but there were no differences among the fava bean fed dogs. These data suggest that fava beans are suitable for inclusion in dog diets, especially if fermented, and that protein intake may play a role in cardiovascular health. In a similar study from the same laboratory, Quilliam et al. sought to investigate whether the fiber brought in by the inclusion of pulses would decrease glycemic response, decrease the rate of digestion, and decrease the bioavailability of macronutrients. Dogs were fed six diets formulated at an inclusion level of 20% available carbohydrates using different ingredients, including legumes, for 7 days. The inclusion of ingredients that possess greater amylose and dietary fiber decreased total tract apparent digestibility, but this was not due to increased bile acid losses. Overall, the authors noticed this decrease in digestibility and the glycemic response was largely due to a lower animal protein content, and that this could put dogs at risk for chronic deficiencies of taurine and may result in the etiology of dilated cardiomyopathy, and that longer-term studies are warranted. In a similar vein, Gizzarelli et al. investigated different carbohydrates sources and their effects on plasma and whole blood taurine status. With diets formulated to provide similar essential nutrients, there was no impact on plasma or whole blood taurine confirming that indeed diets supply nutrients and the ingredients, when used properly, do not affect taurine status. The research into the etiology of dilated cardiomyopathy will continue to be investigated and the characterization of these diets, including detailed nutrient and anti-nutrient content, will continue as the research community and pet food companies continue to pursue new world ingredients as described above.

While cardiac disease may have been recently in the news, overweight and obesity remain the number one nutritionally related issue in our pet population. Niese et al. examined pets and owners both undergoing a simultaneous weight loss program. Overall, the combined results of both human and dog weight loss studies suggest that when either dog or human are actively attempting to lose weight, that it results in passive weight loss in the other and supports using an integrative approach to supporting human and pet weight loss. This opens a new opportunity for physicians and veterinarians to work together and offers a unique integrative or One Health approach touted by the authors. Overall, we expect to see emerging approaches to managing health in our companion animals and the collaboration among human and animal health communities to advance this area of research.

While pet nutrition is riddled with the problems of current formulas, companion animal researchers stay focused on understanding a myriad of arenas regarding nutrient sufficiency. Ruggeiro and Backus compared supplementing cats with either vitamin D2 or 25(OH)D2. Overall, supplementation with 25(OH)D2 was superior to supplementation of vitamin D2 to increase vitamin D status as measured by the presence of vitamin D epimers and may suggest that we need to consider multiple epimeric forms of vitamin D when considering what to supplement in diets. In feline nutrition, we also see a move toward the desire by owners to feed plant-based products, and Dodd et al. report on a case study where a young male cat presents with chronic feline lower urinary tract disease (FLUTD) and was placed on a home-made plant-based diet with the hopes of increasing water intake and promoting an acidic, dilute urine. Indeed, this dietary treatment reduced urinary saturation, and the FLUTD resolved and no subsequent FLUTD was reported by the owner, promoting the use of plant-based diets as an option for the treatment of FLUTD. Indeed, pet owners will continue to drive the industry through their unique consumer trends and, while not always supported by scientists and health care professionals, we need to be open to the communities we serve to understand the perspectives that they hold and the demands they have for the products that companion animal research supports.

## Author Contributions

AS and GB had primary responsibility for the final content. All authors read and approved the final manuscript.

## Conflict of Interest

DC was employed by the company Prairie Swine Centre, Inc. The remaining authors declare that the research was conducted in the absence of any commercial or financial relationships that could be construed as a potential conflict of interest.

## Publisher's Note

All claims expressed in this article are solely those of the authors and do not necessarily represent those of their affiliated organizations, or those of the publisher, the editors and the reviewers. Any product that may be evaluated in this article, or claim that may be made by its manufacturer, is not guaranteed or endorsed by the publisher.
